# Ifi27l2a acts as a cytokine that promotes a pro-inflammatory phenotype in microglia and enhances neuroinflammation

**DOI:** 10.21203/rs.3.rs-7383703/v1

**Published:** 2025-09-24

**Authors:** Sodam Kim, Haven Burrous, Elisabeth Harmon, Lauren Vance, Ting Wu, Alexander Andersohn, Andrea Doan, Darby Wolocko, Zachary Wise, Yuki Kitamura, Joo Eun Jung, Gabriela Colpo, Lucy Couture, Frank Blixt, Louise McCullough, Sean Marrelli, Gab Seok Kim

**Affiliations:** The University of Texas Health Science Center at Houston; The University of Texas Health Science Center at Houston; The University of Texas Health Science Center at Houston; The University of Texas Health Science Center at Houston; The University of Texas Health Science Center at Houston; The University of Texas Health Science Center at Houston; The University of Texas Health Science Center at Houston; The University of Texas Health Science Center at Houston; The University of Texas Health Science Center at Houston; The University of Texas Health Science Center at Houston; The University of Texas Health Science Center at Houston; The University of Texas Health Science Center at Houston; The University of Texas Health Science Center at Houston; The University of Texas Health Science Center at Houston; The University of Texas Health Science Center at Houston; The University of Texas Health Science Center at Houston; The University of Texas Health Science Center at Houston

## Abstract

Interferon alpha-inducible protein 27 like 2A (*Ifi27l2a*) was initially identified as an interferon-stimulated gene (Isg) involved in host-dependent mechanisms during viral invasion. We have recently shown that the Ifi27l2a protein also plays a critical role in microglia and the spreading of neuroinflammation. However, the detailed mechanisms of action of intracellular and extracellular Ifi27l2a in glial cells, particularly in inflammatory and neurodegenerative diseases, are not yet defined. We now report that systemic inflammation leads to an elevated level of circulating Ifi27l2a in the plasma of lipopolysaccharide (LPS)-treated mice, which is associated with elevated *Il1b* mRNA expression in the brain. Elevated IFI27L2 (human isoform) was also found in plasma of human stroke patients. To test whether extracellular (e.g. secreted) Ifi27l2a can contribute as an autocrine or paracrine inducer of microglial activation and inflammation, we treated microglial cells with recombinant Ifi27l2a (rIfi27l2a). Treatment with rIfi27l2a led to increased expression of proinflammatory cytokines and elevated reactive oxygen species (ROS) levels in microglial cells. These effects resulted from alterations in mitochondrial function and a metabolic shift from oxidative phosphorylation toward glycolysis for ATP synthesis. Additionally, Ifi27l2a led to increased caspase-1 activity, demonstrating that Ifi27l2a causes Nlrp3 inflammasome activation. RNA sequencing revealed that Ifi27l2a mediates transcriptional changes reflecting activated microglia. These data support an extracellular role of Ifi27l2a as a novel cytokine, in which it promotes a phenotypic shift in microglia toward a proinflammatory phenotype. Targeting Ifi27l2a may therefore provide an additional strategy to reduce microglia-mediated inflammation in the brain.

## INTRODUCTION

Although various immune cells are involved in neuroinflammation and neurodegeneration in diseased brains, microglia are the primary cells in the central nervous system (CNS) responsible for initiating and propagating inflammation, and eventually contributing significantly to the development of brain pathology and neurodegeneration^1–3^. Increased microglial inflammation is initiated by the detection of the cellular debris and pathogens (bacterial, viruses, or foreign substances) in the brain though pattern recognition receptors (PRRs) like Toll like receptor (TLR), Nod-like receptors (NLR) and intrinsic inflammatory stress produced from immune cells^4–6^. This initial activation transitions microglia into a heightened inflammatory state, characterized by the expression of various inflammatory mediators and reactive oxygen species to perform their innate immune functions^7,8^. In neurodegenerative diseases, altered or excessively activated microglia exhibit a proinflammatory phenotype^9^. These microglia produce and release cytokines and chemokines into the extracellular space, generating a source for autocrine or paracrine signaling, which contributes to neuroinflammation and neurodegeneration^10,11^. However, microglia can also adopt reparative, anti-inflammatory phenotypes characterized by the production of anti-inflammatory mediators, enhanced recognition of cellular debris, and increased digestion enzymes during the phagocytosis process^12–14^. This phagocytic activity helps clear harmful cellular debris and mitigate detrimental inflammation, ultimately contributing to the restoration of neuronal function^15–17^. Therefore, a better understanding of the mechanisms whereby microglial phenotypic changes occur within the context of inflammatory diseases and at different age stages is crucial. The identification of key hub molecules that drive the propagation of inflammation is another opportunity of investigation to further understand disease progression and for developing targeted therapeutic strategies. Consequently, the development of precise tools for time-specific and context-specific modulation of microglia phenotype changes and inhibiting harmful microglial response represents a promising strategy for managing excessive inflammation in diseased brains^2,18^. One suited application of this strategy is for the targeted mitigation of pathological inflammation in the post-stroke brain to help restore brain function and improve neurological outcome^19^.

Emerging lines of evidence underscore the critical role of interferon signaling, particularly type I interferon signaling, in the pathogeneses of neurodegenerative diseases including Alzheimer’s diseases (AD) and stroke^20–23^. Notably, the *sustained* brain inflammation also appears to be driven by the type I interferon signaling pathway. Both acute and chronic interferon-mediated inflammation significantly impact neurological function and contribute to the progression and severity of these diseases^24^. Recent studies demonstrate that interventions targeting type I interferon signaling, such as the deletion of interferon receptor (type I IFN receptors; *Ifnar1* or *Ifnar2*), can mitigate neuroinflammation (post-stroke and amyloid-beta induced) and improve functional outcome^23,25^. These findings support the premise that interferon signaling is a critical pathway for therapeutic strategies seeking attenuate neuroinflammatory responses and mitigate disease progression in neurogenerative conditions^26^. A variety of genes are acutely or chronically expressed in response to interferons in immune cells. To enhance the therapeutic potential of targeting interferon signaling in the inflamed brain, it is now necessary to identify the specific roles of individual interferon signaling pathways, rather than type I interferon signaling as a whole^27,28^. This targeted approach on individual interferon stimulated genes (Isg) will provide a more precise understanding of how interferon-mediated signaling affects neuroinflammation and disease progression^29^.

In our previous study, we demonstrated a pro-inflammatory contribution of upregulated *interferon alpha-inducible protein 27 like 2A* (*Ifi27l2a*) in microglia within the diseased brain^30^. While Ifi27l2a was originally identified as one of the interferon-inducible genes expressed for host defense mechanisms against viral infection^31^, our study revealed that upregulation of *Ifi27l2a* in microglia additionally promotes increased neuroinflammation and worsened functional stroke outcome following ischemic stroke^30^. One component of Ifi27l2a’s deleterious effects is through a triggered switch in mitochondrial function that results in increased ROS production. As a measure of the translational value of targeting Ifi27l2a, we demonstrated that even partial deletion of *Ifi27l2a* (Ifi27l2a^+/−^ mice) reduced brain infarct, lessened gliosis, and improved functional outcome in an experimental model of stroke^30^.

In the present study, we found that the Ifi27l2a protein is elevated in models of systemic inflammation and detectable in the circulating blood. These findings suggested that Ifi27l2a could be released during inflammation, prompting us to ask whether the specific *secretion* of Ifi27l2a might contribute to how this protein spreads inflammation in the brain. We speculated that locally secreted or elevated circulating Ifi27l2a could promote a pro-inflammatory phenotypic change of nearby microglia (or other cell types) and thus propagate brain inflammation in the context of stroke or other brain pathologies. In the present study, we tested the hypothesis that the Ifi27l2a protein can act as a novel cytokine that contributes to the initiation and propagation of inflammation in microglia and other cell types within the brain.

The results of this study provide the first demonstration that Ifi27l2a can function as a novel cytokine that drives microglia to a proinflammatory phenotype. Based on these findings, we propose that targeting the Ifi27l2a protein (such as with small molecules or neutralizing antibodies) may represent a new strategy to reduce microglial-mediated neuroinflammation in the brain following stroke, Alzheimer’s disease (AD), sepsis, or other neurodegenerative diseases.

## MATERIALS AND METHODS

### Mice

C57BL/6 male mice, aged 16–20 months were used for *in vivo* experiments. All animal studies were approved the UTHealth Animal Welfare Committee (approval number: AWC 23–0035). The animals were maintained in an animal room with constant temperature (69–73°F) and humidity (30–70%) and reversed 12-h light/dark cycle. The animals had access to regular rodent chow and tap water ad libitum.

### Systemic inflammation LPS injection model

Lipopolysaccharide (LPS, Escherichia coli 0111: B4, Sigma) was dissolved in PBS and administered to mice intraperitoneally (i.p.) at a dose of 5 mg/kg. Twenty-four hours after the injection, blood samples were collected to measure the level of Ifi27l2a protein in plasma. At the same time, brains were harvested for the assessment of CNS inflammation by expression of Il-1β mRNA (qRT-PCR).

### Plasma isolation and measurement of Ifi27l2a protein levels

For plasma isolation, blood was collected by heart puncture into heparinized tubes, centrifuged, and the supernatant was saved as plasma. Ifi27l2a protein plasma levels were measured by ELISA (Abbexa, Cambridge, UK).

### Human plasma collection

All participants were admitted to Memorial Hermann Hospital, Houston, TX, USA, with a diagnosis of acute ischemic stroke. All patients provided written informed consent before completing the study. All procedures were approved by the Institutional Review Board at The University of Texas Health Science Center at Houston, Houston, Texas, USA, (IRB: HSC-MS-17–0452).

### Human IFI27L2 ELISA

Peripheral blood was collected in sterile vacutainers and transported on ice for processing. Blood plasma was isolated by centrifuging samples at 1,200 × g for 10 min at 4°C, followed by plasma supernatant isolation and further centrifugation at 10,000 × g for 10 min at 4°C. Samples were stored at −80 °C in aliquots until analysis. Plasma samples were used to quantify the levels of IFI27L2 (human isoform of Ifi27l2a) by Enzyme-Linked Immunosorbent Assay (ELISA) kit according to the procedures supplied by the manufacturer (abx527820, Abbexa, Cambridge, UK).

### Cell culture

SimA9 cells, an immortalized mouse microglial line cells, were purchased from ATCC (#CRL-3265, Manassas, VA, USA). These cells were maintained in Dulbecco’s Modified Eagle Medium/F12 (DMEM/F12, #11320082, Thermo Fisher Scientific Inc., Waltham, MA, USA), supplemented with 10% fetal bovine serum (FBS, #10082147, Thermo Fisher) and 5% horse serum (HS, #H1138, Millipore Sigma, Burlington, MA, USA). Additionally, bEnd.3 cells, a microvascular endothelial cell line derived from mouse brain, were cultured in DMEM/F12 supplemented with 10% FBS and 100 μg/mL penicillin/streptomycin (#15140122, Thermo Fisher). Both SimA9 and bEnd.3 cells were passaged and utilized for up to 5 passages. The cells were cultured in a humidified incubator with 5% CO_2_ at 37°C.

### Mouse microglia cell culture

Primary microglial cultures were established following previously described methods^32^. Neonatal mice (mixed sexes) were euthanized in accordance with institutional animal care guidelines. Whole brains were extracted from postnatal day 1–2 from Ifi27l2a^+/+^ and Ifi27l2a^+/−^ mice, both on a C57Bl/6J background. The olfactory bulb and cerebellum were excised, and the meninges and hippocampal region were removed. The remaining brain tissue was transferred to ice-cold HBSS. Cortical tissues were digested with 0.25% trypsin-EDTA for 15 minutes at 37°C, followed by the addition of FBS. The digested tissues were centrifuged at 1000 rpm for 5 minutes, and the pellet was resuspended in DMEM medium (#11995073, Thermo Fisher) supplemented with 20% FBS and 100 μg/mL penicillin/streptomycin. The cell suspension was filtered through a 70-μm nylon cell strainer. Subsequently, the cell suspension was seeded at a density of one brain per 75 cm³ flask and maintained at 37°C in a 5% CO_2_ incubator for 7 days.

Mixed glial cultures were maintained for 10 to 14 days, with a complete medium change every three days. Upon reaching 80% confluence, microglia were isolated by tapping to dislodge the microglia from the astrocytic monolayer. The isolated microglia were then centrifuged at 1000 rpm for 5 minutes, resuspended in a microglia-specific medium consisting of DMEM supplemented 20% FBS, 100 μg/mL penicillin/streptomycin, 10 ng/mL recombinant mouse M-CSF Protein (#416-ML-050, R&D Systems, Minneapolis, MN, USA), and subsequently plated onto poly-D-lysine-coated plates.

### Detection of ROS using CellROX^®^ Deep Red

To facilitate the live detection of reactive oxygen species (ROS) production, Sim-A9 cells were seeded and treated with rIfi27l2a (0, 10, 100 ng/ml) or vehicle (final con. 0.0001% BSA in media) for 24 hours. Following treatment, the culture medium was replaced with serum-free DMEM/F12. At the start of the staining procedure, CellROX^®^ Deep Red (#C10422, Thermo Fisher) was introduced at a final concentration of 5 μM and incubated for 30 minutes at 37°C, shielded from light. The medium was then removed, and the cells were washed three times with PBS. The quantification of ROS was analyzed using CytoFLEX flow cytometry (Beckman Coulter, Brea, CA, USA).

### ECAR and OCR Analysis / Measurement of cellular bioenergetics by Seahorse assays

An XF96 Extracellular Flux Analyzer (Seahorse Bioscience) was used for real-time analyses of the extracellular acidification rate (ECAR) and oxygen consumption rate (OCR) in Sim-A9 cells and primary microglia, following the manufacturer’s protocol (Agilent Technologies, Santa Clara, CA, USA). In brief, the cells were seeded in an XF 96-well microplate containing complete medium and incubated overnight at 37°C. Subsequently, the cells were treated with Ifi27l2a protein (or vehicle) in serum-free medium for 24 hours. The XF Calibrant was utilized to hydrate the XFp cartridges, which were then incubated overnight at 37°C. On the following day, the cell medium was replaced with pre-warmed pH 7.4 XF DMEM-based assay medium supplemented with 25 mM glucose, 2 mM glutamine, and 2 mM pyruvate (Agilent).

The Mito stress test was conducted on the Sim-A9 cells treated with Ifi27l2a protein (or vehicle) for 24 hours using the Seahorse XF Cell Mito Stress Test Kit (#103015–100, Agilent). OCR and ECAR were measured under basal conditions, followed by the sequential addition of 1.5 μM oligomycin (Port A), 2 mM FCCP (Port B), and 0.5 mM Rotenone/antimycin A (Rot/AA, Port C). This protocol facilitates the precise calculation of OCR associated with basal respiration, maximal respiration, ATP production, and non-mitochondrial respiration.

The ATP rate assay was conducted on the Sim-A9 cells treated with Ifi27l2a protein (or vehicle) for 24 hours using the Seahorse XF Real-Time ATP Rate Assay Kit (#103592–100, Agilent). In this assay, both OCR and ECAR were measured under basal conditions, followed by the sequential addition of 1.5 μM oligomycin (Port A) and 0.5 mM rotenone/antimycin A (Rot/AA, Port B). This protocol facilitates the precise calculation of OCR to analyze the parameters such as glycoATP and mitoATP production rate, total ATP production rate, as well as the percentage of glycolysis and oxidative phosphorylation, respectively.

The glycolysis stress test was conducted on the Sim-A9 cells treated with Ifi27l2a protein (or vehicle) for 24 hours using the Seahorse XF Glycolysis Stress Test Kit (#103020–100, Agilent). ECAR was measured under basal conditions, followed by sequential injections of 10 mM glucose, 1.5 μM oligomycin, and 100 mM 2-deoxyglucose (2-DG). This procedure facilitates the estimation of extracellular acidification attributable to non-glycolytic acidification, glycolysis, and glycolytic capacity.

The background was subtracted from all data, and the values were normalized to the cell viability, which was established at 100% in the control group, utilizing Wave Pro software Version 10.2.0 (Agilent). The results were subsequently plotted using GraphPad Prism 10 software. The seahorse experiments were repeated three times and presented as the data from three independent experiments.

### Cell viability assay

Cell viability was measured by Thiazolyl Blue Tetrazolium Bromide (MTT) assay. In brief, following the appropriate treatment of the cells, the culture media was discarded. A solution of MTT (#5655, Millipore Sigma) was then added to each well at concentration of 0.5mg/ml and incubated at 37°C for 2 hours. After the incubation period, the MTT solution was removed, and isopropanol was added to each well. The absorbance was measured at 570 nm using a microplate reader (EnSpire^®^ Multimode Plate Reader, PerkinElmer, MA, USA).

### RNA isolation and quantitative real-time quantitative polymerase chain reaction (qPCR)

Total RNA was extracted from the cells using the RNeasy kit (Qiagen, Hilden, Germany) and the Pury RNA Plus kit (#P2030–050, GenDEPOT, TX, USA), following the manufacturer’s protocols. Complementary DNA (cDNA) was synthesized from mRNA utilizing the Iscript^™^ Reverse Transcription Supermix (#1708841, Bio-Rad, Hercules, CA, USA). The qPCR reactions consisted of the source cDNA, specific primer pairs for each target gene, and the PCR SYBR Green Supermix (#1725271, Bio-Rad). The expression levels of the following mouse genes were examined: *Ifi27l2a, Il-1α, Il-1β, Tnfα, Mmp2, Mmp3, Mmp9, Acod1, Icam1, Vcam1, Tf, and Mcp1*. Each quantitative gene expression reaction for the target genes was conducted in a final volume of 20 μl and analyzed using the CFX96/384 Touch Real-Time PCR Detection System (Bio-Rad). Gene expression curves were evaluated on a logarithmic scale, and threshold cycles were calculated mathematically. The quantitative expression of the target genes was determined using the 2^−ΔΔCT^ method, with normalization for experimental variability across multiple steps, utilizing the levels of the housekeeping gene, glyceraldehyde-3-phosphate dehydrogenase (GAPDH). All reactions were performed in triplicate. The primers used in this study are listed in the supplemental table 1.

### FLICA caspase 1 activity assay

This assay utilizes the far-red fluorescent probe 660-YVAD-FMK to label the active caspase-1 enzyme within the cells. Sim-A9 cells were cultured in a well plate and subsequently treated with Ifi27l2a protein or vehicle. After a 24-hour incubation period, the active caspase-1 levels were quantified using a Pyroptosis/Caspase-1 Assay kit (SKU: 9158, Antibodies Inc., Davis, CA, USA), following the manufacturer’s instructions. In brief, the cells were washed once with PBS and detached by using 0.25% trypsin (T4049, Millipore Sigma). Cells were then resuspended in culture medium, and a working solution of FLICA 660 was added at a final concentration of 60X (at a ratio of 1:60). Cells were gently mixed and incubated at 37°C for one hour in the absence of light. During the incubation period, the cells were resuspended by gently swirling every 20 minutes to maintain an even distribution of FLICA, since cells otherwise settle at the bottom of the tubes. Upon completion of the incubation, 1X Cellular Wash Buffer was added and mixed gently. The samples were centrifuged at 200g for 10 minutes at room temperature (RT), and the supernatant carefully aspirated (performed twice). Cells were then resuspended in 300 μl of 1X Cellular Wash Buffer and placed on ice before quantitative analysis by flow cytometry (CytoFLEX flow cytometer, Beckman).

### Western blot

Whole cell proteins were extracted from SimA9 cells utilizing RIPA Cell Lysis Buffer (#R4100010, GenDEPOT) supplemented with a protease inhibitor cocktail (#11836170001, Sigma-Aldrich). The extraction process was conducted on ice and incubated for 30 minutes on an orbital shaker. The resulting protein lysates were quantified using the Pierce BCA Protein Assay Kit (#23209, Thermo Fisher). Equal quantities of protein were run on a sodium dodecyl sulfate–polyacrylamide gel electrophoresis (SDS-PAGE) using a gradient gel (4–20%) at a voltage of 80 V for 2 hours, followed by transfer onto polyvinylidene fluoride (PVDF) membranes (#1620177, Bio-Rad) for 1.5 hours at 80 V. The PVDF membrane was blocked with 3% nonfat dry milk in Tris-buffered saline (TBS) containing 0.1% Tween-20 (T-PBS) at room temperature for 1 hour, and then incubation with primary antibodies was performed overnight at 4°C. The membrane was then washed with T-TBS and incubated with horseradish peroxidase (HRP)-conjugated secondary antibodies at room temperature for 1 hour. Positive signals were visualized using Western ECL Substrate (#1705061, Bio-Rad) with the LI-COR Odyssey^®^ M apparatus (LI-COR Biosciences, Lincoln, NE, USA). The intensities of the protein bands were normalized against actin as a loading control (#ab207674, Abcam, Cambridge, UK). The following primary antibodies were used at the following dilutions: Gasdermin-D (1:1000, ab209845, Abcam) and Il-1β (1:1000, BAF401, R&D Systems).

### RNA sequencing and DESeq2 analysis

RNA samples were submitted to the Cancer Genomics Center at The University of Texas Health Science Center at Houston (CPRIT RP180734). Total RNA was quality-checked using Agilent RNA 6000 Pico kit (#5067 − 1513) by Agilent Bioanalyzer 2100 (Agilent Technologies, Santa Clara, USA). RNA with an integrity number of greater than 7 was used for library preparation. Libraries were prepared with NEBNext Poly(A) mRNA Magnetic Isolation Module (E7490L, New England Biolabs), NEBNext Ultra II Directional RNA Library Prep Kit for Illumina (E7760L, New England Biolabs) and NEBNext Multiplex Oligos for Illumina (E6609S, New England Biolabs) following the manufacturer’s instructions. The quality of the final libraries was examined using Agilent High Sensitive DNA Kit (#5067 − 4626) by Agilent Bioanalyzer 2100 (Agilent Technologies, Santa Clara, USA), and the library concentrations were determined by qPCR using Collibri Library Quantification kit (#A38524500, Thermo Fisher Scientific). The libraries were pooled evenly and underwent paired-end 150-cycle sequencing on an Illumina NovaSeq System (Illumina, Inc., USA). The count matrix was obtained for the RNAseq data sets using the Rosalind web tool (ROSALIND, Inc., San Diego, CA) and then fed into DESeq2 in R for analysis. To conduct a comprehensive gene ontology analysis, 100 upregulated differentially expressed genes (DEGs) identified in rIfi27l2a-treated microglia (vs. control) were input into g:Profiler^33^. Additionally, Gene Set Enrichment Analysis (GSEA) was performed using GSEA 4.3.3^34^ to identify significantly enriched molecular function categories. A normalized count matrix generated from DESeq2 was used as input data. Gene sets were obtained from the Gene Ontology Molecular Function (GO MF) collection. Analysis parameters were set to 1,000 permutations to assess statistical significance. Enrichment plots were generated to visualize the distribution of genes from each significantly enriched gene set. The normalized enrichment score (NES) was used to compare enrichment results across gene sets, with positive NES values indicating enrichment in rIfi27l2a-treated microglia and negative values indicating enrichment in the control (Veh) group.

### TCA, Glycolysis, and Itaconate Analysis using LC-MS

Metabolites were extracted from cell pellets of Sim-9A cells treated with rIfi27l2a (or vehicle) for 24 hours, with pool samples used as the quality control, following the previously described extraction procedure^35–38^. Metabolites related to the TCA cycle, glycolysis, and their intermediates were separated using a Luna 3 μM NH2 (100 Å) HPLC column (Phenomenex, Torrance, CA), with 5 mM ammonium acetate in water (pH 9.9) and acetonitrile as the mobile phase solvents. Itaconate was separated using an ACQUITY UPLC HSS T3 column (1.8 μm, 2.1 × 150 mm, Waters Corporation, Milford, MA), with solvent A consisting of water containing 0.1% formic acid and solvent B consisting of acetonitrile containing 0.1% formic acid. Metabolite separation was performed using an Agilent 1290 Infinity HPLC system, and data were acquired using an Agilent 6495B Triple Quadrupole mass spectrometer (Agilent Technologies, Santa Clara, CA) in Multiple Reaction Monitoring (MRM) mode with negative ionization^37,38^. The acquired data were analyzed using Agilent Mass Hunter Quantitation software, with thorough manual checking of integration and evaluation of all peaks across samples. Data were normalized using spiked isotopically labeled internal standards and subsequently log2-transformed. Differential metabolites were identified by applying the Benjamini-Hochberg method with a false discovery rate (FDR < 0.25) correction following a Student’s t-test.

### Statistical data analysis

Statistical data analysis was performed using Prism 10 (GraphPad Software, San Diego, CA, USA) with p < 0.05 considered statistically significant. Data are presented as the mean ± standard error of the mean (SEM) and analyzed using an unpaired t-test for two-group comparisons, or a one-way (or two-way) ANOVA with post-hoc tests for multiple comparisons, depending on whether there was one factor (oneway) or two factors (two-way). p < 0.05 was defined as statistically significant (* p < 0.05; ** p < 0.01; *** p < 0.001).

## RESULTS

### Ifi27l2a is released from cells in inflammatory conditions.

**Figure 1:**
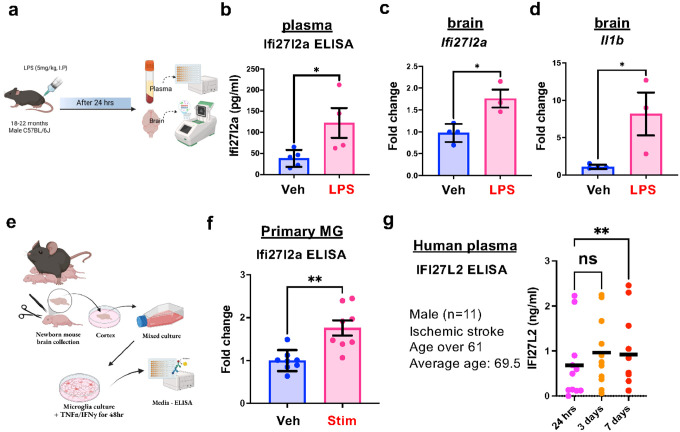
Circulating Ifi27l2a is increased in inflammatory situation. **(a)** Endotoxemia was induced in aged male mice by i.p. injection of LPS (5 mg/kg). Plasma was collected 24 hours post-injection from mice that had been injected with either PBS or LPS. **(b)** ELISA was performed to measure plasma Ifi27l2a protein levels. LPS treatment resulted in an increase in plasma Ifi27l2a levels in mice (n=4–5, Student’s t-test, * p<0.05 compared to Veh). **(c, d)** Brains were harvested and RNA isolated from the brains were used for qRT-PCR to measure the level of *Ifi27l2a* and *Il1b* mRNA (n=3–4, Student’s t-test, * p<0.05 compared to Veh). **(e, f)** Ifi27l2a was released in primary microglia after TNFα/IFNγ (20 ng/ml) for 48 hours treatment (n=8, Student’s t-test, ** p<0.01, Veh vs. Stim). **(g)** The IFI27L2 plasma level of human stroke patients at 24 hours, 3 days and 7 day after onset of stroke (n=11, male patients, black solid lines indicate the mean values, ** p<0.01 compared to 24-hour time point, repeated measures one-way ANOVA with Sidak’s multiple comparisons)

We used a systemic inflammation model to determine whether inflammatory conditions lead to increased Ifi27l2a protein that can then be detected in the blood. Endotoxemia was induced in aged mice (male, 18–22 month old) by administering LPS (5 mg/kg, i.p.) to induce inflammation in the periphery and brain parenchyma (Fig. 1a). Twenty-four hours post-injection of LPS or vehicle, we measured plasma Ifi27l2a levels by ELISA. LPS-mediated inflammation led to a 3.2-fold increase in plasma Ifi27l2a compared to PBS-treated mice (122.1+/−35.3 vs. 38+/−8.9 pg/ml) (Fig. 1b, n = 4–5, * p < 0.05). Parallel measurements of brain mRNA showed that *Ifi27l2a* and inflammatory marker gene, *Il1b*, were significantly increased following LPS injection, validating the potential of LPS to promote increased expression of both *Ifi27l2a* and a classic marker of neuroinflammation (Fig. 1c, d, n = 3–4, * *p* < 0.05).

While it is known that *Ifi27l2a* expression can be triggered by inflammatory stress, no prior reports describe if or how the Ifi27l2a protein might be released. As a first step to explore the potential role for Ifi27l2a as a cytokine, we used primary microglial cultures to determine if Ifi27l2a protein could be released into the media following proinflammatory cytokine treatment. Primary microglia were treated with pro-inflammatory cytokines (TNF-α, 20 ng/mL and IFN-γ, 20 ng/mL) to induce microglial inflammation and subsequent *Ifi27l2a* expression^39^. After 48 hours of treatment, we collected the media and performed ELISA to measure the concentration of Ifi27l2a (Fig. 1e). Following pro-inflammatory cytokine treatment, the media showed a 1.7+/−0.17 fold increase in Ifi27l2a protein (Fig. 1f, n = 7, ** p < 0.01), supporting the capacity of immune cells to release Ifi27l2a in response to inflammatory stress. To investigate the temporal dynamics of the human isoform (IFI27L2) during stroke-induced inflammation, we analyzed serial plasma samples collected from the patients at three time points: 24 hours, 3 days, and 7 days post-stroke onset. This cohort consisted of male patients over 61 years of age (mean age 69.5 years, n = 11). IFI27L2 levels showed significant elevation at both 3 and 7 days compared to the initial 24-hour time point, indicating a progressive increase in expression during the acute inflammatory phase following stroke (Fig. 1g). Taken together, these *in vivo*, *in vitro* and human data indicate that Ifi27l2a protein can be released from microglia (and possibly other cells) during inflammatory signaling and further support a pathophysiological role of *extracellular* Ifi27l2a protein in the context of brain or systemic inflammation.

### Extracellular Ifi27l2a stimulates a pro-inflammatory phenotype in microglia.

**Figure 2:**
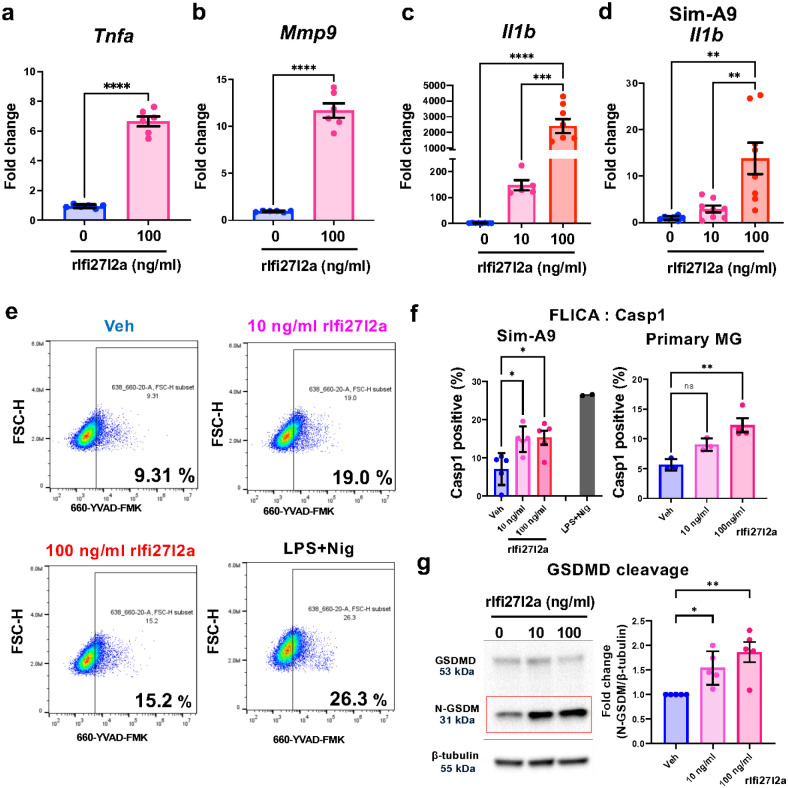
Ifi27l2a alone initiates microglial activation and induces proinflammatory gene expression and inflammasome activation. Primary microglia and Sim-A9 cells were treated with rIfi27l2a was added to cells for 24 hours. **(a)** *Tnfa* and **(b)** *Mmp9* mRNA levels were measured by qRT-PCR 24 hours after Ifi27l2a treatment (n=6, **** p<0.000, Student t-test). **(c)**Inflammatory marker gene, *Il1b* was evaluated 24 hours after Ifi27l2a treatment in primary microglia (n=5–8, *** p<0.001, ****p<0.0001, compared to 0 ng/ml, One-way ANOVA with Tukey’s multiple comparisons). **(d)** *Il1b*mRNA levels in Sim-A9 cells after rIfi27l2a treatment were measured (n=7–8, ** p<0.01 compared to 0 ng/ml, One-way ANOVA with Tukey’s multiple comparisons). Caspase-1 assay using the FLICA probe was performed with Sim-A9 cells treated with rIfi27l2a for 24 hours. **(e)** Representative images of gating strategy for identifying FLICA positive cells (cells with active-Casp1). LPS+Nigericin were used for positive control. **(f)** The percentage of FLICA-positive cells was increased with rIfi27l2a treatment (n=3, * P<0.05, ** p<0.01, One-way ANOVA with Tukey’s multiple comparisons). **(g)** western blotting of GSDMD showed increased cleaved GSDMD (N-terminal fragment) after rIfi27l2a treatment in Sim-A9 (n=5, * p<0.05, ** p<0.01, One-way ANOVA with Tukey’s multiple comparisons).

Based on our findings that the Ifi27l2a protein can be released and detected in blood plasma following inflammatory stimuli, we speculated that Ifi27l2a may act as a pro-inflammatory cytokine. We hypothesized that Ifi27l2a can stimulate MG in an autocrine or paracrine manner, thereby contributing to the further spread of inflammation. Therefore, we generated recombinant Ifi27l2a (rIfi27l2a) to investigate whether treatment with extracellular rIfi27l2a can mimic the characteristics of a pro-inflammatory cytokine (i.e. initiate inflammation in microglia). With extracellular application of rIfi27l2a, primary MG demonstrated significant upregulation of proinflammatory marker genes, *Tnfa*, *Mmp9*, and *Il1b* mRNA (Fig. 2a, b, c). Application of rIfi27l2a to Sim-A9 cells also showed upregulation of *Il1b* mRNA (Fig. 2d). Experiments examining the effect of rIfi27l2a on *Il1b* expression in primary MG and Sim-A9 further showed a dose-dependent effect. Note that rIfi27l2a did not alter cell viability in primary microglia (at 10 or 100 ng/ml) (**Supplementary Fig. 1a**, n = 4); however, it did increase overall RNA amount (an indirect indicator of microglial activation) (**Supplementary Fig. 1b**, n = 6, ** p < 0.01). These data demonstrate that extracellular Ifi27l2a acts as a proinflammatory signaling molecule on microglia.

### Extracellular rIfi27l2a promotes inflammasome activation in microglia.

Aberrant Nlrp3 inflammasome activation leads to inflammation in immune cells and causes subsequent pyroptosis, a form of immune cell death mediated by caspase-1 with inflammasome complex. This inflammasome complex cleaves the pro-I1–1b, pro-Il-18 and Gasdermin D (GSDMD) to their mature forms and facilitates the release of toxic proinflammatory mediators from the cells through molecular pores^40–42^. Thus, detecting caspase-1 activity is a reliable tool for assessing Nlrp3-meditated inflammasome activation and subsequent pyroptosis in microglia. We utilized a recently developed dye in order to directly measure caspase-1 activity. This FLICA/casp1 is a fluorescently-labeled peptide substrate for caspase-1, which specifically binds to the active site of caspase-1 when active site is opened (i.e. indicator of active caspase-1). As a positive control, treatment with LPS and Nigericin significantly increased inflammasome activation in Sim-A9 cells (Fig. 2e). Extracellular treatment with rIfi27l2a alone (10 and 100 ng/ml) also increased the percent of the cells demonstrating active caspase-1 activity (Fig. 2e, f, * p < 0.05, ** p < 0.01). Note that primary MG also demonstrated increased caspase-1 activity in response to rIfi27l2 treatment (Fig. 2f). Lastly, N-terminal of Gasdermin D (N-GSDM), which is a form of GSDMD cleaved by caspase-1, was significantly increased with rIfi27l2a treatment in Sim-A9 cells (Fig. 2g, * p < 0.05, ** p < 0.01). These data highlight the potential for extracellular Ifi27l2a to promote Nlrp3 inflammasome activation in microglia.

### Extracellular rIfi27l2a promotes pro-inflammatory transcriptional changes in microglia (RNAseq).

**Figure 3:**
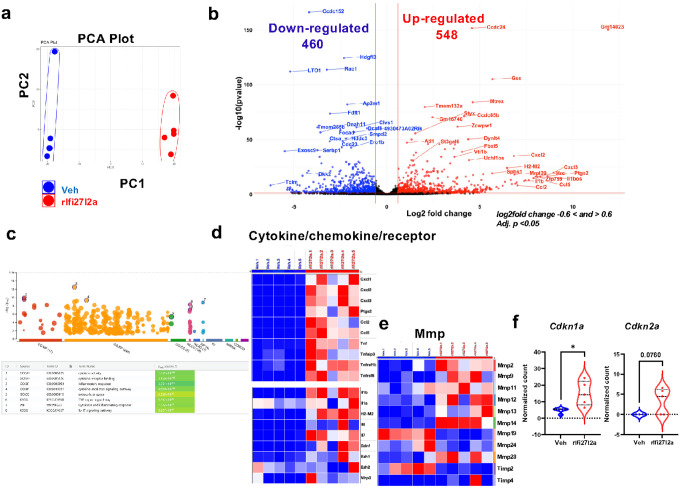
Transcriptomic changes in primary microglia following rIfi27l2a stimulation. Primary microglia treated with either vehicle or 100 ng/ml of rIfi27l2a were used for RNA sequencing. **(a)** The PCA plot shows distinct transcriptomic profiles between the vehicle and rIfi27l2a-treated cells (n=5 per group). **(b)** 460 down-regulated genes and 548 up-regulated gene by rIfi27l2a were identified (cutoff: log2fold change −0.6< and >0.6, adj. p <0.05). Volcano plot revealed the highly differentially expressed genes, including *Cxcl2*, *Cxcl3*, *Ptgs2*, *Il7*, *Tnf*, *Ccl2*, and *Il6*. **(c)** Gene ontology (GO) analysis using g:Profiler reveals that differentially expressed genes (DEGs) altered by Ifi27l2a treatment are involved in several key biological processes and cellular pathway. **(d)** Heatmap shows increased expression of inflammatory genes, including genes for proinflammatory cytokines, chemokines, receptors. **(e)** Heatmap for Mmps. **(f)**Violin plots show an increased expression pattern of genes related to cellular senescence such as *Cdkn1a* (* p<0.05) and *Cdkn2a*(p=0.076).

To more comprehensively explore transcriptional changes of microglia treated with extracellular Ifi27l2a, we treated primary microglia with rIfi27l2a at 100 ng/ml for 24 hrs and performed RNAseq (n = 5, each group). The PCA plot comparing the vehicle (Veh) and rIfi27l2a groups revealed significantly different gene expression profiles (Fig. 3a). Using DESeq2, we next identified a total of 1,008 differentially expressed genes (DEGs) between treated groups. Among these DEGs, 548 genes were significantly upregulated and 460 genes were significantly downregulated, with a significance threshold of an adjusted p-value of 0.05 and absolute log2FoldChange of 0.6 (cutoffs of −0.6: down-regulated and 0.6: up-regulated) (Fig. 3b). The volcano plot clearly shows the upregulation of a set of proinflammatory genes such as *Cxcl3*, *Ptgs2*, *Ccl5*, *Cxcl2, Tnf, Mmp9*, and *Il1b* (Fig. 3b). To decode signaling pathways that could be initiated by Ifi27l2a, we conducted a comprehensive gene ontology (GO) analysis with 100 upregulated DEGs in rIfi27l2a-treated microglia compared to control using g:Profiler. GO analysis revealed the involvement of several key biological processes, including cytokine activity (GO:MF:0005125, adjusted p-value = 1.23×10^− 10^), cytokine receptor binding (GO:MF:0005126, adjusted p-value = 3.16×10^− 10^), inflammatory response (GO:BP:0006954, adjusted p-value = 3.35×10^− 13^), cytokine-mediated signaling pathways (GO:BP:0019221, adjusted p-value = 4.97×10^− 10^), IL-17 signaling pathway (KEGG:04657, adjusted p-value = 8.82×10^− 8^), and TNF signaling pathway (KEGG:04668, adjusted p-value = 5.462×10^− 9^) (Fig. 3c). Additionally, the analysis indicated involvement in cytokines and inflammatory responses (WP:222, adjusted p-value = 1.72×10^− 9^) and processes that occur in the extracellular space (GO:CC:0005615, adjusted p-value = 4.56×10^− 6^). Additional GSEA analysis using DESeq2 normalized count matrix revealed gene sets associated with the following GO molecular functions: ‘chemokine receptor binding,’ ‘cytokine activity,’ ‘cytokine receptor binding,’ ‘antigen binding,’ ‘chemokine activity,’ and ‘hormone binding’ (**Supplementary Fig. 2**).

Heatmaps further reveal that rIfi27l2a induces multiple classic proinflammatory genes and receptors (Fig. 3d) and Mmps (Fig. 3e). Interestingly, rIfi27l2a also caused marked induction of *Cdkn1a*, a senescence marker gene (Fig. 3f, * p < 0.05). Another senescence marker gene, *Cdkn2a*, neared statistical significance (p = 0.076). These combined data indicate that extracellular Ifi27l2a can significantly modulate microglial phenotype, promoting a gene expression consistent with inflammation and senescence. Furthermore, GO analyses are highly supportive of our proposed role of Ifi27l2a as a proinflammatory cytokine.

### Exposure to extracellular Ifi27l2a protein promotes increased ROS production and loss of mitochondrial membrane potential.

**Figure 4:**
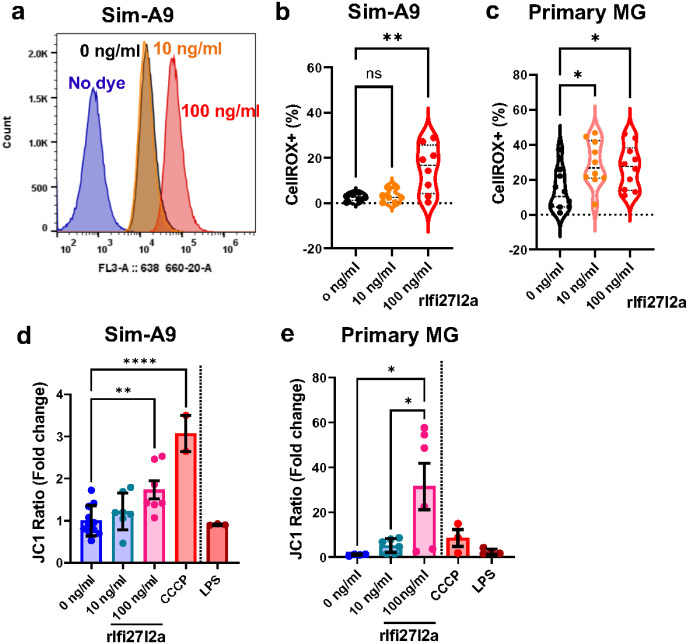
Ifi27l2a itself causes loss of mitochondrial membrane potential and increases ROS. **(a)** ROS levels were measured using CellRox ROS detection dye in Sim9A cells after Ifi27l2a treatment (blue: no dye, black: 0 ng/ml, yellow: 10 ng/ml, red: 100 ng/ml). **(b)** The percentage of CellRox positive cells was increased by rIfi27l2a in Sim-A9 cells (n=6–8. One-way ANOVA with Dunnett’s multiple comparisons test, ** p <0.01, compared to 100 ng/ml Ifi27l2a). **(c)** The percentage of CellRox positive cells was increased in primary MG after rIfi27l2a treatment (n=10, One-way ANOVA with Holm-Sidak’s multiple comparisons test, * p<0.05, compared to 10 and 100 ng/ml Ifi27l2a). **(d)**rIfi27l2a treatment caused a reduction in mitochondrial (Mt) membrane potential measured by JC1 dye in Sim-A9 cells. JC1 dye was utilized to assess Mt membrane potential following 24-hour treatment with rIfi27l2a. CCCP was used as a positive control and LPS was used as a negative control (n=3–8, One-way ANOVA with Tukey’s multiple comparisons test, * p<0.05 vs 0 ng/ml rIfi27l2a). **(e)** The loss of mitochondrial membrane potential in primary MG by rIfi27l2a (n=4–6, * p<0.05, One-way ANOVA with Tukey’s multiple comparisons test).

We recently demonstrated that Ifi27l2a is critically involved in spreading neuroinflammation after stroke and that overexpression of Ifi27l2a in unstressed microglia is sufficient to promote ROS production and cell activation^39^. However, the specific effects of *released* Ifi27l2a resulting from inflammatory stress on microglia have not been reported. Therefore, to examine the effect of exogenous Ifi27l2a on microglial activation, we treated cells with different concentrations of recombinant Ifi27l2a protein (rIfi27l2a) and measured the effects on mitochondrial function. We first evaluated Sim-A9 cells (a microglial like cell line) treated with rIfi27l2a at 0, 10 and 100 ng/ml for 24 hours and measured intracellular ROS production with CellRox red dye. Treatment with 100 ng/ml rIfi27l2a resulted in a significant increase in ROS levels compared to 0 ng/ml (veh) and “no dye control” (Fig. 4a, b, n = 6–8, ** p < 0.01, 0 ng/ml vs. 100 ng/ml). Treatment with 10 ng/ml rIfi27l2a was not statistically different from the 0 or 100 ng/ml rIfi27l2a groups. Next, using primary microglia, we demonstrated significantly increased ROS production with both 10 and 100 ng/ml rIfi27l2a (Fig. 4c, n = 10, * p < 0.05).

ROS can be elevated due to the functional changes in mitochondria in inflammatory conditions^43^. Thus, we also measured the changes in mitochondrial (Mt) membrane potential using JC1 dye. Sim-A9 cells were treated with rIfi27l2a (0, 10, and 100 ng/ml) or LPS (20 ng/ml) for 24 hours. CCCP was used as a positive control for inducing the loss of mitochondrial membrane potential^44^. The ratio of JC1 aggregate (Red) to JC1 monomer (Green) is indicative of Mt membrane potential loss (Green/Red) and was detected by flow cytometry. Interestingly, even in the absence of other stimuli, rIfi27l2a (100 ng/ml) caused significant reduction in Mt membrane potential (Fig. 4d, n = 7–11, ** p < 0.05, 0 ng/ml vs. 100 ng/ml rIfi27l2a). In contrast, treatment with LPS (20 ng/ml) did not alter Mt membrane potential. Treatment of primary MG with rIfi27l2a produced a similar pattern, but with a greater reduction of Mt membrane potential with 100 ng/ml of rIfi27l2a (Fig. 4e, n = 4–6, * p < 0.05).

### Extracellular Ifi27l2a induces altered mitochondrial function in microglia.

**Figure 5:**
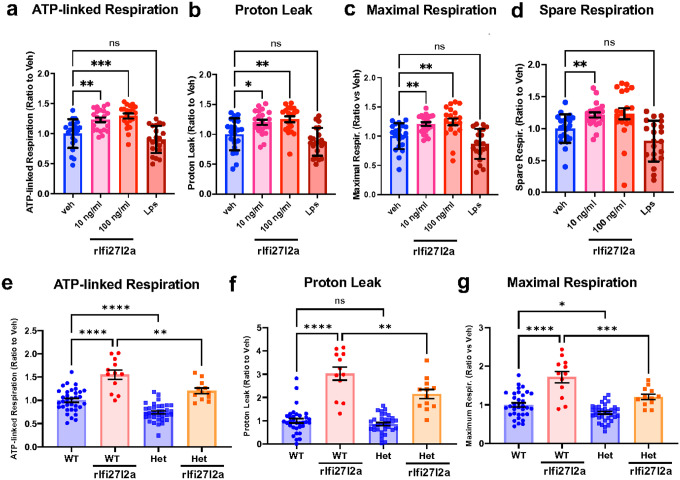
Ifi27l2a modulates mitochondrial function. Sim-A9 cells were seeded onto a Seahorse plate, and rIfi27l2a was added to cells for 24 hours. Mitochondrial function was then assessed using Seahorse cell analysis. **(a)** ATP-linked respiration, **(b)** proton leak, (**c**) maximal respiration, and (**d**) spare respiration were calculated using Seahorse mito stress kit. Data were expressed as a ratio to veh. LPS were used for control (n=20–21, one-way ANOVA with Dunnett’s T3 multiple comparisons, * p<0.05, ** p<0.01, *** p<0.001 compared to Veh). Primary microglia from WT vs Het (Ifi27l2a^+/−^) were seeded onto a Seahorse plate, and rIfi27l2a was added to cells for 24 hours. Mitochondrial function was then assessed using Seahorse cell analysis. **(e)** ATP-linked respiration, **(f)** proton leak, and **(g)** maximal respiration were calculated using Seahorse mito stress kit. Data were expressed as a ratio to WT with no rIfi27l2a (n=12–34, Two-way ANOVA with Tukey’s multiple comparisons, * p<0.05, ** p<0.01, *** p<0.001).

Given our finding that rIfi27l2a promotes increased ROS levels and loss of mitochondrial membrane potential in microglia, we next asked whether extracellular Ifi27l2a also affects mitochondrial respiration. For this, we used the Seahorse mito stress assay to assess the effect of rIfi27l2a on mitochondrial oxygen consumption rate (OCR). Treatment with rIfi27l2a in Sim-A9 cells caused an increase in ATP-linked respiration compared to vehicle control (Fig. 5a, n = 10–21, ** p < 0.01, *** p < 0.001). This effect was not observed in the cells treated with LPS. Proton leak, an indicator of mitochondrial damage, was also increased by rIfi27l2a treatment, but not by LPS (Fig. 5b, * p < 0.05, ** p < 0.01). Additionally, an increase in maximal respiration and spare respiration was observed with rIfi27l2a at 10 ng/ml and 100 ng/ml (Fig. 5c, d). These data indicate that signaling via extracellular Ifi27l2a can modulate mitochondrial function, specifically resulting in altered mitochondrial respiration.

To further investigate the mechanism by which extracellular Ifi27l2a increases mitochondrial stress, we isolated primary microglia from WT and *Ifi27l2a*^+/−^ mice (Het mice) and conducted a Seahorse mitochondrial stress assay. Het mice were used to assess the potential contribution of *intracellular* Ifi27l2a in the response to application of the extracellular protein. Similar to the response in Sim-A9 cells, rIfi27l2a increased ATP-linked respiration, proton leak, and maximum respiration in WT microglia, indicating that extracellular Ifi27l2a induces mitochondrial stress in primary MG (Fig. 5e, f, g). Interestingly, in Het microglia, measures of rIfi27l2a-induced mitochondrial stress (ATP-linked respiration, proton leak, and maximum respiration) were significantly diminished compared to WT microglia (Fig. 5e, f, g, * p < 0.05, ** p < 0.01, *** p < 0.001, **** p < 0.0001, two-way ANOVA with Tukey’s multiple comparisons). These findings suggest that intracellular Ifi27l2a signaling contributes to the mechanism by which extracellular Ifi27l2a promotes increased true mitochondrial stress in microglia.

**Figure 6:**
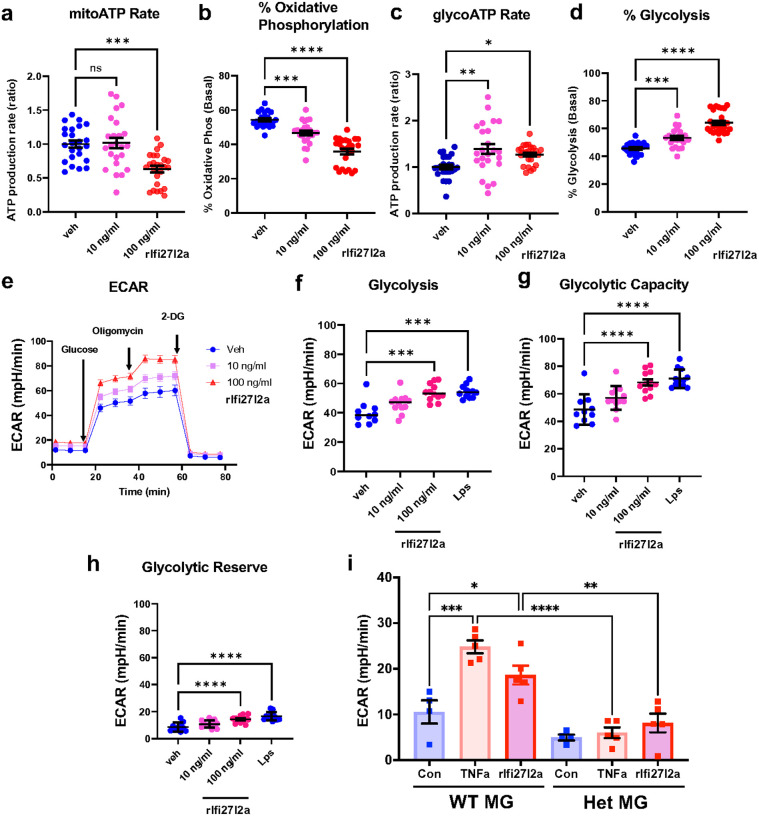
Ifi27l2a shifts metabolic pathway to glycolysis for ATP production. Sim-A9 cells were seeded onto a Seahorse plate and treated with rIfi27l2a for 24 hours. Mitochondrial function was then assessed using Seahorse cell analysis. **(a)** mitoATP production rate and **(b)** percentage of oxidative phosphorylation were measured using Seahorse ATP rate kit (n=24–25, **** p<0.0001, one-way ANOVA with Tukey’s multiple comparisons test). **(c)** GlycoATP production rate and (**d**) percentage of glycolysis were also assessed using Seahorse ATP rate kit (n=24–25, **** p<0.0001, one-way ANOVA with Tukey’s multiple comparisons test). Glycolysis was further evaluated using Seahorse glycolysis stress kit. **(e)** ECAR levels were measured following glucose, oligomycin and 2-DG injection in Ifi27l2a-stimulated cells (n=12). **(f)**Glycolysis, **(g)**glycolytic capability, and **(h)**glycolytic reserve were increased in Ifi27l2a-stiumlated cells compared to vehicle-treated cells (n=10–12, *** p<0.001. One-way ANOVA with Dunnett’s multiple comparisons test). Primary microglia from WT brains and Het brains were seeded onto a Seahorse plate and treated with TNFα or rIfi27l2a for 48 hours. **(i)** Glycolysis was then assessed using Seahorse cell analysis (n=4–5, * p<0.05, ** p<0.01, *** p<0.005, **** p<0.0001. one-way ANOVA with Tukey’s multiple comparisons).

The proinflammatory phenotype of microglia is associated with a metabolic shift toward glycolysis^45^. Therefore, to specifically examine whether extracellular Ifi27l2a can promote this proinflammatory metabolic shift, we measured the glycolysis rate in Sim-A9 cells following rIfi27l2a incubation. The Seahorse ATP rate assay revealed a rIfi27l2a-mediated reduction in mitoATP production rate and % of oxidative phosphorylation (Fig. 6a, b), as well as increased glycoATP production rate and % of glycolysis (Fig. 6c, d). Using the glycolysis stress kit, we demonstrated that extracellular rIfi27l2a treatment also increased ECAR, glycolysis, glycolytic capacity, and glycolytic reserve (Fig. 6e, f, g, h). LPS, which is known to enhance the glycolysis rate in microglia and immune cells, was used as a positive control and to contextualize the magnitude of the rIfi27l2a response. Together, these findings reveal that extracellular Ifi27l2a can drive a metabolic shift from oxidative phosphorylation to glycolysis, consistent with its proposed overall role in promoting a proinflammatory microglial phenotype. We confirmed the increased glycolysis in primary MG treated with TNF*α* or rIfi27l2a (Fig. 6i). Moreover, the increase in glycolysis was significantly reduced in Het MG (Fig. 6i), suggesting that exogenous Ifi27l2a drives glycolysis via intracellular Ifi27l2a-mediated signaling.

### Extracellular Ifi27l2a promotes upregulation of glycolysis-related genes in microglia (RNAseq) and alters TCA cycle metabolites.

**Figure 7:**
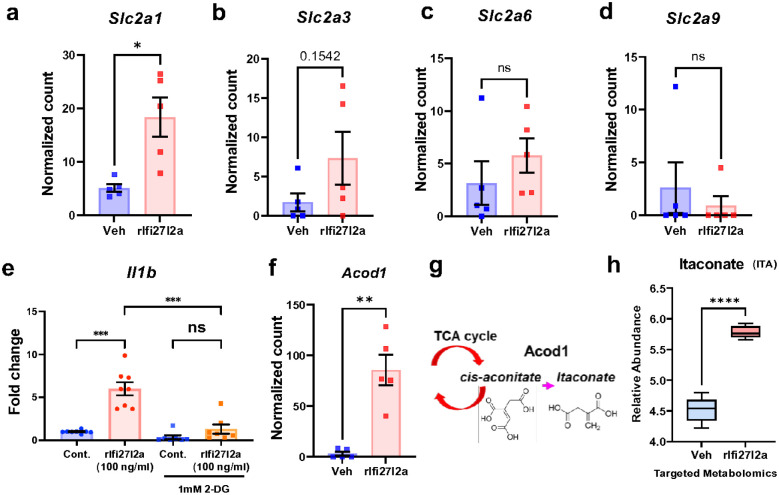
Involvement of metabolic changes in glycolysis in Ifi27l2a-mediated proinflammatory response. Primary microglia treated with either vehicle or 100 ng/ml of rIfi27l2a were used for RNA sequencing. The genes related to glucose transferred was increased by rIfi27l2a. **(a)** Slc2a1- Glut1, **(b)** Slc2a3- Glut3, **(c)** Slc2a6, **(d)** Slc2a9. **(e)** 2-DG, inhibitor of glycolysis reduced the expression of *Il1b* in rIfi27l2a treated Sim9A (n=7–8, ** p<0.01, *** p<0.001, One-way ANOVA with Sidak’s multiple comparisons test). **(f)** *Acod* (a gene encoding the enzyme involved in glycolysis/TCA cycle) expression was significantly increased in Ifi27l2a-treated microglia (** p< 0.01, Student t-test). **(g, h)** Itaconate, a metabolite produced through the action of Acod1, was increased in cells treated with rIfi27l2a (n=3–5, ** p<0.01, Student t-test).

Focusing our RNAseq analysis on glycolysis-relevant gene expression, we found that *Slc2a1* (encodes glucose transporter 1) was upregulated following rIfi27l2a treatment (Fig. 7a, * p < 0.05). Two other glucose transporter isoforms showed a non-significant trend toward upregulation (*Slc2a3* and *Slc2a3*), whereas *Slc2a9* was unchanged (Fig. 7b, c, d). To investigate the involvement of glycolysis in the potentiated proinflammatory response by rIfi27l2a, we used 2-deoxy-D-glucose (2-DG), an inhibitor of the glycolytic process to assess the requirement for glycolysis on rIfi27l2a-mediated gene transcription. 2-DG is structurally similar to glucose and is taken up by glucose transporters, where it competes with glucose for entry into the glycolysis pathway^46^. Interestingly, the rIfi27l2a-induced increase in *Il1b* expression was abolished by 2-DG treatment, indicating that glycolysis is required for the enhanced *Il1b* expression by rIfi27l2a (Fig. 7e, n = 7–8, *** p < 0.001). The Ifi27l2a-mediated elevation of *Mmp2* and *Mmp9* expression was not downregulated by 2-DG (**Supplementary Fig. 3a, b**), suggesting that glycolysis-independent mechanisms are involved in the regulation of *Mmp* expression in microglia. We also identified a gene related to glycolysis and the TCA cycle, *Acod1*, among the top upregulated genes by extracellular rIfi27l2a (Fig. 7f, n = 5, ** p < 0.01). The *Acod1* gene codes for the Irg1 protein, which converts cis-aconitate to itaconate during the TCA cycle (Fig. 7g). Itaconate has been shown to have immune-modulatory function. Therefore, to test if the Ifi27l2a-mediated increase in *Acod1* results in increased itaconate production, we measured itaconate in Sim-A9 cells treated with extracellular rIfi27l2a or vehicle. With rIfi27l2a treatment, itaconate was significantly elevated (Fig. 7h, n = 3–5, ** p < 0.01), indicating the potential of extracellular Ifi27l2a to modify TCA cycle function and increased itaconate production.

### Metabolites in glycolysis and the TCA cycle were altered by rIfi27l2a.

**Figure 8:**
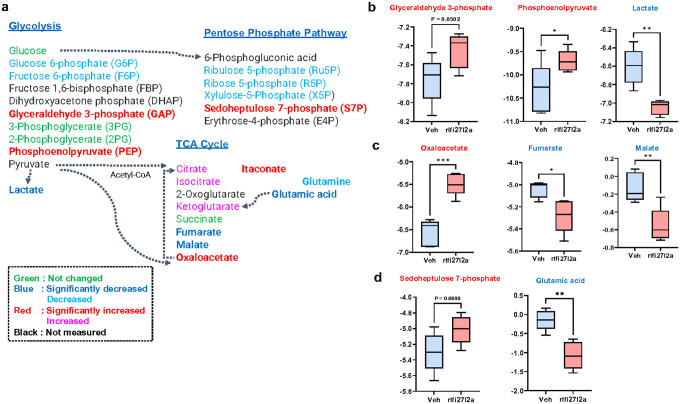
The levels of metabolites were altered by rIfi27l2a. **(a)** Summary of microglial metabolite levels in glycolysis, pentose phosphate pathway, and TCA cycle, measured by targeted metabolomics (n = 5 per group). **(b)**Intracellular metabolite levels of glyceraldehyde 3-phosphate, phosphoenolpyruvate, and lactate in glycolysis (n=5 per group, * p < 0.05, ** p < 0.01, two-tailed unpaired t-test). **(c)**Intracellular metabolite levels of oxaloacetate, fumarate and malate in TCA cycle (n=5 per group, * p < 0.05, ** p < 0.01. *** p < 0.001, two-tailed unpaired t-test). (d) Intracellular metabolite levels of sedoheptulose 7-phosphate and Glutamic acid in TCA cycle (n=5 per group, ** p < 0.01, two-tailed unpairedt-test).

Since we observed the regulatory effect of rIfi27l2a on the glycolysis and TCA cycle pathways, we then assessed the levels of metabolites produced in the glycolysis process and TCA cycle by using targeted metabolomics approaches. Figure 8a summarizes the metabolite changes induced by rIfi27l2a treatment in Sim-A9 cells, showing elevated intracellular metabolites of glycolysis (such as glyceraldehyde 3-phosphate and phosphoenolpyruvate), the pentose phosphate pathway (sedoheptulose 7-phosphate), and the TCA cycle (oxaloacetate). In the metabolites of glycolysis, we found that rIfi27l2a increased the cellular levels of glyceraldehyde 3-phosphate and phosphoenolpyruvate. However, the level of lactate was reduced in Ifi27l2a-treated cells compared to vehicle-treated cells (Fig. 8b). In TCA cycle metabolites, the oxaloacetate level was significantly increased by rIfi27l2a. However, the levels of fumarate and malate were markedly downregulated in rIfi27l2a-treated cells (Fig. 8c). Sedoheptulose 7-phosphate, a major metabolite in the pentose phosphate pathway, was upregulated, while the glutamic acid level was significantly downregulated in rIfi27l2a-treated cells (Fig. 8d). This targeted metabolomic data indicates that rIfi27l2a induces alterations in metabolic pathways in microglia, particularly impacting the glycolysis-TCA pathway.

### Proinflammatory effect of extracellular Ifi27l2a on endothelial cells.

We have demonstrated that rIfi27l2a induces microglial activation, as evidenced by increased *Il1b* expression *in vitro*, suggesting potential paracrine effects of released Ifi27l2a on other cells within the neurovascular unit. To explore this potential, we examined whether extracellular Ifi27l2a could activate endothelial cells. First, we assessed the ability of rIfi27l2a to cause stress fiber formation in cultured endothelial cells. Stress fibers can form in endothelial cells in response to inflammatory cues and ischemic stress. Mouse bEnd.3 endothelial cells were treated with rIfi27l2a (100 ng/ml) for 24 hours and then stained with phalloidin to evaluate stress fiber formation. Increased stress fibers (typical of actin stress fiber morphology) were produced in response to rIfi27l2a treatment (Supplementary Fig. 4a, b, n = 9–11, ** p < 0.01). Extracellular rIfi27l2a also induced upregulation of proinflammatory cytokines (*Il1a* and *Il1b*) and genes associated with coagulation *(TF*), and cell adhesion (*Icam1* and *Mcp1*) (**Supplementary Fig. 4c, d**). *Mmp3* and *Mmp9* levels in endothelial cells were also increased by rIfi27l2a, whereas *Mmp2* levels remained unchanged. (**Supplementary Fig. 5**). These findings highlight the potential for Ifi27l2a to act as a cytokine contributing to endothelial cell activation and potential cerebrovascular dysfunction.

### Extracellular Ifi27l2a promotes cell death of primary neurons.

Given the capacity of microglia to release Ifi27l2a during inflammatory stress, neurons represent another potential target of extracellular Ifi27l2a. We therefore investigated the potential neurotoxic effect of Ifi27l2a on primary neurons cultured from E17 rat embryos. After 14 days in vitro (DIV), we exposed primary neurons to an inflammatory stimulus (LPS, 100 ng/ml) and co-treated with either rIfi27l2a or vehicle for 24 hours. The amount of resulting cell death was quantified with the LDH assay. LPS alone induced a slight non-significant increase of LDH release into the media. However, co-treatment with rIfi27l2a demonstrated a significant enhancement of LDH release a dose-dependent manner (10 and 100 ng/ml) (**Supplementary Fig. 6**, n = 5–6, ** p < 0.01). These findings support the potential of released Ifi27l2a to exacerbate neuronal cell death under inflammatory conditions.

## DISCUSSIONS

In this study, we explored the proposed novel role of Ifi27l2a as a secreted or released factor in the propagation of inflammation. In microglia, we defined the effects of extracellular Ifi27l2a protein in inducing inflammation via ROS generation, mitochondrial metabolism alteration, and Nlrp3 inflammasome activation. Our data also suggested that extracellular Ifi27l2a induced some degree of pyroptosis, a type of cell death triggered by excessive inflammation in immune cells. We further examined how released Ifi27l2a could affect other cell types in the brain and found that extracellular Ifi27l2a induced proinflammatory effects in endothelial cells and increased vulnerability to inflammatory stimuli in neurons. Together, the presented data supports the criteria for Ifi27l2a to be recognized as a proinflammatory cytokine.

### Ifi27l2a acts as a cytokine.

To be classified as a cytokine, a molecule must be produced by immune cells and have an effect on other cells within immune system, thereby influencing the immune response^47–49^. Cytokines are typically small proteins or peptides (5–25 KDa) that function as signaling molecules via autocrine, paracrine, or endocrine mechanisms. Cytokines are not generally constitutively expressed, but rather they are induced by certain stimuli (e.g. injury, inflammatory stress, etc.). Ifi27l2a satisfies these criteria and characteristics of cytokines, as Ifi27l2a is a small protein that is significantly induced in microglia in response to various inflammatory challenges, is released from microglia and promotes proinflammatory signaling in microglia (autocrine and paracrine) or other cell types in the brain (paracrine). In addition, Ifi27l2a was elevated in blood plasma during an experimental model of LPS-induced inflammation as well as in the plasma obtained from human stroke patients, suggesting that Ifi27l2a may additionally contribute to broader inflammation throughout the body (i.e. endocrine mechanism). From our studies, we could not determine if extracellular Ifi27l2a acted through a specific receptor or via other signaling mechanisms. Future efforts will be required to identify possible Ifi27l2a receptors or membrane-associated binding molecules in basal or inflammatory conditions.

### Ifi27l2a as a modifier of mitochondrial metabolic changes.

We examined mitochondrial energetics programed by extracellular Ifi27l2a in microglia using Seahorse cell analyzer by measuring OCR and ECAR levels in real-time. With the Seahorse XF Analyzer, we have revealed interesting aspects of Ifi27l2a on mitochondrial function, though we still need to determine whether this action is a direct effect on mitochondria or an indirect effect through intracellular signaling pathways from a yet-to-be-identified receptor for Ifi27l2a. Our data show that Ifi27l2a can function as a modifier of mitochondrial activity to actual ATP production in mitochondria via oxidative phosphorylation, impacting ATP production from mitochondria and cellular metabolism. Our finding of increased ATP-linked respiration, as measured by the Mito Stress Test, implies that Ifi27l2a-stressed microglia may attempt to meet ATP demands for proper functionality by enhancing mitochondrial respiration capability. However, despite this increased ATP-linked respiration in mitochondria, the ATP Rate Kit revealed a decrease in mitochondrial ATP (mitoATP) production rate in a dose-dependent manner with rIfi27l2a treatment. This decrease may result from inefficiencies of ATP synthase during oxidative phosphorylation due to proton leak (which we observed when Ifi27l2a was treated on microglia), leading to inefficiency of ATP production per unit of oxygen consumed. This findings suggest that mitochondrial proton leakage in Ifi27l2a-treated microglia negatively affects mitochondrial respiration and leads to uncoupling from actual ATP production in mitochondria. As a result, microglia adapt by shifting their metabolic ATP production pathway from oxidative phosphorylation to glycolysis to compensate the loss of inefficient ATP production from OXPHOS. In this context, microglia compensate for reduced mitochondrial energetics by preferentially utilizing glycolysis for ATP production, resulting in a higher glycolytic rate and an increased percentage of ATP derived from glycolysis. Overall, this transition induced by rIfi27l2a could help microglia maintain ATP levels despite diminished efficiency of oxidative phosphorylation in order to convert microglia to the proinflammatory phenotype. The Ifi27l2a-induced metabolic conversion from OXPHOS to glycolysis could lead to a more profound proinflammatory response that is demanded for proper microglial activation and function^45^. This hypothesis is further supported by our data, which show that 2-DG, a glycolysis inhibitor, diminished Ifi27l2a-induced proinflammatory gene expression. These findings emphasize the significance of glycolysis in ATP production as a key mechanism driving proinflammatory responses induced by extracellular Ifi27l2a.

Altered glycolysis has been observed in many immune cells, such as T cells and macrophages, especially when challenged by inflammatory cues and in the tumor microenvironment. These cells require a high demand for ATP over a short period to exhibit inflammatory changes in gene expression and cell behavior. This phenomenon, similar to events known as the Warburg effect, highlights Ifi27l2a-meditated shift towards glycolysis for energy production. Thus, our findings regarding the role of Ifi27l2a are closely linked to the events occurring in activated immune cells and cancerous cells. This metabolic shift by Ifi27l2a helps transform microglia into a form fully prepared for optimal microglial function in an oxygen poor proinflammatory environment.

### Extracellular Ifi27l2a causes mitochondrial alternations and pyroptosis.

The induction of increased proton leak suggests that extracellular Ifi27l2a signaling causes some amount of mitochondrial dysfunction related to uncoupling from actual ATP production, resulting in increased ROS generation and a metabolic shift toward glycolysis. The proposed mitochondrial functional alteration is supported by elevated ROS levels and the loss of mitochondrial membrane potential using JC1 dye observed with rIfi27l2a treatment.

We have found that Ifi27l2a induces a degree of pyroptosis, as evidenced by increased Caspase-1 activity and cleaved Gasdermin D, which forms pores in the membrane. Through these pores, secreted proteins can be released during the pyroptosis process. The NLRP3 inflammasome complex functions as both a cytosolic DNA sensor and an executor for cleaving substrates such as pro-Il1b, pro-Il18, and the Gasdermin family protein. To activate the NLRP3 inflammasome pathway, sensor proteins, Nlrp3 must detect cytosolic DNA or mitochondrial DNA (Mt DNA) leaked to the cytosol from dysfunctional mitochondria. Based on our data, we speculate that Ifi27l2a may ultimately promote some amount of mitochondrial DNA leakage into the cytosol due to mitochondrial membrane potential loss. However, it remains to be demonstrated experimentally whether Ifi27l2a directly causes inflammasome activation and mitochondrial dysfunction in parallel, or if mitochondrial dysfunction induced by Ifi27l2a leads to inflammasome activation and then pyroptosis.

We have demonstrated that, without any other inflammatory stress, extracellular Ifi27l2a can induce pyroptosis by activating the inflammasome and executing the final steps of the pyroptotic process. Specifically, inflammasome activation leads to Gasdermin D cleavage, which is a critical final step in pyroptosis. While we observed the cleaved form of Gasdermin D, we did not measure pore formation. Through these pores, IL-1β, IL-18, and other Gasdermin D pore-associated secreted proteins could be released, warranting further investigation on how Ifi272la is involved in true Gasdermin D (GSDMD) pore formation.

### Extracellular Ifi27l2a increases glycolysis and TCA cycle metabolites.

Glycolysis and the TCA cycle are tightly connected and an increase in glycolysis leads to more pyruvate production, which results in more acetyl-CoA entering the TCA cycle, potentially enhancing TCA cycle flow and ATP production. We observed a significant reduction in lactate levels, which is a unique feature of extracellular Ifi27l2a compared to other cytokines and inflammatory mediators. This suggests that most metabolites produced during glycolysis upon Ifi27l2a stimulation are entering the TCA cycle, leading to an increase in the levels of itaconate and oxaloacetate. Additionally, our metabolomics data suggest that the increased metabolites in glycolysis and the TCA cycle may be due to the upregulation of glucose transporters, Glut1 and Glut3 and elevated glucose uptake. An upregulation of *Glut1* and *Glut3* was also detected in our RNAseq dataset. However, whether extracellular Ifi27l2a stimulation results in enhanced glucose uptake remains to be studied.

While we demonstrated the induction of predictable proinflammatory genes in Ifi27l2a-treated microglia, our RNAseq data also revealed significant induction of an unanticipated gene, *Acod1*. The *Acod1* gene codes for an enzyme (Irg1) involved in the TCA cycle, which converts cis-aconitate to itaconate. While we have not yet determined the contribution of this enzyme to Ifi27l2a-induced glycolysis, ATP production, and proinflammatory response, we have found that the metabolite, itaconate, was significantly increased in cells treated with Ifi27l2a. The effects of itaconate (anti-inflammatory vs. proinflammatory) are cell- and context-dependent. Therefore, future experiments are needed to elucidate the consequences of elevated itaconate in activated microglia to determine if itaconate enhances or attenuates the inflammatory response triggered by Ifi27l2a signaling and/or other inflammatory mediators.

In summary, we provide evidence that the Ifi27l2a protein can act as a novel cytokine. In microglia, extracellular Ifi27l2a induces mitochondrial energetic alterations (reduction of mitochondrial membrane potential, ATP production, ROS generation, metabolic shift to glycolysis) and proinflammatory gene expression, leading to the activation of the NLRP3 inflammasome. Extracellular Ifi27l2a also influences other cell types, including cell types within the neurovascular unit (e.g. endothelial cells and neurons). The consequences of Ifi27l2a-mediated signaling in microglia, endothelial cells, and neurons are predicted to include propagation of brain inflammation, blood brain barrier dysfunction, and increased neuronal death. Thus, targeting the extracellular Ifi27l2a protein (e.g. small molecules or neutralizing antibodies) could serve as an effective intervention for mitigating microglial-mediated neuroinflammation in the brain following stroke, Alzheimer’s disease (AD), sepsis, or other neurodegenerative diseases.

## Supplementary Material

Supplementary Files

This is a list of supplementary files associated with this preprint. Click to download.

- Supplementaryinformation.docx
